# Microfluidic Based Organ-on-Chips and Biomedical Application

**DOI:** 10.3390/bios13040436

**Published:** 2023-03-29

**Authors:** Qiushi Li, Zhaoduo Tong, Hongju Mao

**Affiliations:** 1 State Key Laboratory of Transducer Technology, Shanghai Institute of Microsystem and Information Technology, Chinese Academy of Sciences, Shanghai 200050, China; 2 Center of Materials Science and Optoelectronics Engineering, University of Chinese Academy of Sciences, Beijing 100049, China

Organ-on-a-Chip is a microfluidic cell culture device manufactured via microchip fabrication methods. The device contains continuously perfused chambers with a multi-cellular layer structure, tissue interface, physicochemical microenvironment, and human vascular circulation. It can also be considered a cell culture microengineering device that can mimic and reconstruct the physiological functions of human organs. Microfluidic technology refers to the precise manipulation of fluids in micron-sized structures, which can integrate the basic operations of biochemical experiments, such as sample reaction, preparation, separation, and detection into a tiny chip, with various advantages such as high sensitivity, high integration, high throughput, and high efficiency. In the biomedical field, it can be used in drug synthesis and analysis, in vitro diagnostics, bionic skin tissue and organs, single cell analysis, and nucleic acid analysis. In drug analysis, organ-on-chips possess improved abilities to mimic physiological conditions, contributing to the assay's cost reduction, integration, and miniaturization. Generally, in single-component drug analysis, microfluidic chips have the advantages of miniaturization of assays, rapidity, and low consumption of sample reagents. In more complex multi-component drug analysis, in-line enrichment techniques are often combined to improve detection sensitivity. This Special Issue, “Microfluidic Based Organ-on-Chips and Biomedical Application”, includes four research articles covering epithelium–capillary interface chip, lung chip, microfluidic cell co-culture chip, and surface-fabrication of fluorescent hydroxyapatite, as well as one review article on microfluidic organ-on-a-chip system. The reported microfluidic organ-on-chips were used to detect human soft tissue inflammation, evaluate EGFR-targeted anti-tumor drugs, monitor interactions between macrophages and fibroblasts, and image cancer cells.

The organ-on-a-chip device combines microfabrication and tissue engineering to replicate human organs’ critical physiological environment and function [1]. Thus, it can predict drug responses and environmental effects on organs. Microfluidics enables high-precision control of microscale reagents. As a result, microfluidics has been widely used in organ-on-a-chip systems to simulate a specific organ or multiple organs in vivo [1]. These models integrated with various sensors show great potential in affecting the human environment.

The gingival epithelial–capillary interface is a unique feature of periodontal soft tissue, maintaining homeostasis within the periodontal tissue and preventing the entry of microorganisms and toxic substances into the subepithelial tissue. However, in periodontitis, the function of the interface is disturbed, and the mechanisms of interface disruption are not fully understood. A microfluidic epithelial–capillary barrier with a thin culture membrane (10 µm) was developed to address these limitations, closely mimicking the in vivo gingival epithelial barrier with an immune microenvironment [2]. To validate the established gingival epithelial barrier model, epithelial–capillary interface chips were cultured with human gingival epithelial cells (HGECs) and human vascular endothelial cells (HUVEC), respectively. Their fundamental properties were tested using light microscopy, transepithelial/endothelial electrical resistance (TEER), and permeability analysis. The limitations of this study include using HUVEC cells instead of periodontal endothelial cells derived from periodontal microvasculature. Importantly, this work recapitulates key functional interfaces of periodontal soft tissues and suggests applications in the oral cavity, providing a new in vitro platform for other oral and related organ diseases.

Lung cancer is the most common cancer in the world. Studies have shown that non-small cell lung cancer (NSCLC) accounts for 85% of all lung cancer types, and the overall epidermal growth factor receptor (EGFR) mutation rate is 50.2% [3]. The existing two-dimensional models are inadequate in accurately simulating the physiological characteristics of the lung. Therefore, a lung chip has been developed using 3D print technology with polydimethylsiloxane (PDMS) to detect different EGFR-targeted drugs [3]. A microporous membrane separates the upper and lower channels of the chip, and the upper medium is inoculated with lung cancer cells. In contrast, the lower track is inoculated with vascular endothelial cells and continuously perfused with a cell culture medium. This lung chip can mimic the microenvironment of lung tissue and enable the co-culture of two types of cells at different levels [3]. Two-dimensional well plates were compared with 3D chips, and the result showed that 3D lung chips were superior to 2D well plates in assessing the effects of other EGFR-targeting drugs (gefitinib, afatinib, and osimertinib) on tumor cells; in addition, the results were more consistent with available clinical data [3]. Lung microarrays are necessary for evaluating EGFR-targeted drugs, personalized diagnosis, and pharmacodynamic evaluation.

Macrophages and fibroblasts are two important cells in wound healing. A microfluidic chip consisting of two layers of co-cultures was developed to study the interrelationship between these two cells for exploring wound-healing mechanisms and drug development. Air valves were used to separate fibroblasts from simulating the trauma-healing microenvironment. The fusion rate of fibroblasts with different macrophages in the co-culture system was investigated to reflect the role of other macrophages in trauma healing. The results showed that m2-type macrophages could promote the activation and migration of fibroblasts, which could facilitate the trauma-healing process [4]. Cell migration and cell–cell interactions are critical in tumor formation, embryonic development, and other biological processes.

Hydroxyapatite (HAP) materials are widely used in biomedical materials because of their excellent properties, stable performance, low cost, good biocompatibility, and biodegradability. An efficient fluorescent nanosystem for cell imaging and drug therapy has been developed based on polyethyleneimine (PEI) and functionalized HAP delivered via simple physical adsorption [5]. The HAP nanorods were first functionalized using sodium riboflavin phosphate (HE) to give fluorescent properties based on a ligand exchange process. Next, PEI was attached to the surface through electrostatic attraction to functionalized HAP. The nanosystem can be rapidly taken up by NIH-3T3 fibroblasts and successfully applied for cell culture imaging [5]. In addition, the in vitro release results of Adriamycin hydrochloride (DOX) containing HAP-HE@PEI at high loading showed that DOX released the maximum amount of the drug at pH 5.4 (31.83%), significantly higher than that at pH 7.2 (9.90%), meaning that it can be used as a drug delivery tool. Finally, printing with GelMA using HAP-HE@PEI as 3D inkjet printing ink showed good biocompatibility in the 3D cell culture of RAW 264.7 macrophages [5]. The strategy is based on physical encapsulation (charge attraction), which is simple, fast, efficient, and can occur under reasonably mild conditions.

Organ-on-a-chip technology has good application prospects because of its high similarity to human physiological characteristics. When combined with microfluidics, organ-on-a-chip enables precise manipulation of environmental conditions and simulation of fluidic microenvironments. Considering the general trend of gradually reducing animal trials, microfluidic-based organ-on-a-chip technology will receive more attention and be further developed.
